# A Smartphone-Based Intervention With Diaries and Therapist Feedback to Reduce Catastrophizing and Increase Functioning in Women With Chronic Widespread Pain. Part 2: 11-month Follow-up Results of a Randomized Trial

**DOI:** 10.2196/jmir.2442

**Published:** 2013-03-28

**Authors:** Ólöf Birna Kristjánsdóttir, Egil A Fors, Erlend Eide, Arnstein Finset, Tonje Lauritzen Stensrud, Sandra van Dulmen, Sigrid Hørven Wigers, Hilde Eide

**Affiliations:** ^1^Department of Behavioral Sciences in MedicineFaculty of MedicineUniversity of OsloOsloNorway; ^2^Institute of NursingFaculty of HealthOslo and Akershus University College of Applied SciencesOsloNorway; ^3^Department of Psychiatry; National Competence Centre for Complex Symptom DisordersSt Olav University HospitalTrondheimNorway; ^4^Department of Public Health and General PracticeFaculty of Medicine, General Practice Research UnitNorwegian University of Science and TechnologyTrondheimNorway; ^5^Driv DigitalTønsbergNorway; ^6^NIVEL (Netherlands Institute for Health Services Research)UtrechtNetherlands; ^7^Department of Primary and Community CareRadboud University Nijmegen Medical CentreNijmegenNetherlands; ^8^Faculty of Health SciencesBuskerud University CollegeDrammenNorway; ^9^Jeløy Kurbad Rehabilitation CentreMossNorway

**Keywords:** Internet-based personalized feedback, widespread chronic pain, fibromyalgia, pain management, eHealth, smartphone, Internet, cognitive therapy, catastrophization

## Abstract

**Background:**

Internet-based interventions are increasingly used to support self-management of individuals with chronic illnesses. Web-based interventions may also be effective in enhancing self-management for individuals with chronic pain, but little is known about long-term effects. Research on Web-based interventions to support self-management following participation in pain management programs is limited.

**Objective:**

The aim is to examine the long-term effects of a 4-week smartphone-intervention with diaries and therapist-written feedback following an inpatient chronic pain rehabilitation program, previously found to be effective at short-term and 5-month follow-ups.

**Methods:**

140 women with chronic widespread pain, participating in a 4-week inpatient rehabilitation program, were randomized into two groups: with or without a smartphone intervention after the rehabilitation. The smartphone intervention consisted of one face-to-face individual session and 4 weeks of written communication via a smartphone, consisting of three diaries daily to elicit pain-related thoughts, feelings, and activities, as well as daily personalized written feedback based on cognitive behavioral principles from a therapist. Both groups were given access to an informational website to promote constructive self-management. Outcomes were measured with self-reported paper-and-pencil format questionnaires with catastrophizing as the primary outcome measure. Secondary outcomes included daily functioning and symptom levels, acceptance of pain, and emotional distress.

**Results:**

By the 11-month follow-up, the favorable between-group differences previously reported post-intervention and at 5-month follow-up on catastrophizing, acceptance, functioning, and symptom level were no longer evident (*P*>.10). However, there was more improvement in catastrophizing scores during the follow-up period in the intervention group (M=-2.36, SD 8.41) compared to the control group (M=.40, SD 7.20), *P*=.045. Also, per protocol within-group analysis showed a small positive effect (Cohen’s *d*=.33) on catastrophizing in the intervention group (*P*=.04) and no change in the control group from the smartphone intervention baseline to 11-month follow-up. A positive effect (Cohen’s *d*=.73) on acceptance was found within the intervention group (*P*<.001) but not in the control group. Small to large negative effects were found within the control group on functioning and symptom levels, emotional distress, and fatigue (*P*=.05) from the intervention baseline to the 11-month follow-up.

**Conclusion:**

The long-term results of this randomized trial are ambiguous. No significant between-group effect was found on the study variables at 11-month follow-up. However, the within-group analyses, comparing the baseline for the smartphone intervention to the 11-month data, indicated changes in the desired direction in catastrophizing and acceptance in the intervention group but not within the control group. This study provides modest evidence supporting the long-term effect of the intervention.

**Trial Registration:**

Clinicaltrials.gov NCT01236209; http://www.clinicaltrials.gov/ct2/show/NCT01236209 (Archived by WebCite at http://www.webcitation.org/6FF7KUXo0)

## Introduction

Chronic widespread pain (CWP) is a common cause of suffering in the adult population, with reported prevalence rates between 4% and 10% [1-5]. In addition to pain, fatigue, sleep disturbance, and emotional distress are common [1,5]. A subgroup has more severe symptoms and meets the diagnosis criteria of fibromyalgia [2,5]. Knowledge of the pathogenesis of CWP and fibromyalgia is still evolving; dynamic processes including biological, social, and psychological factors are known to be involved [6]. Multidimensional rehabilitation is the recommended treatment, including interventions based on cognitive behavioral therapy (CBT) where patients learn how thoughts, beliefs, and feelings can influence the pain experience and functioning [6,7]. The short-term effects are well established, but concerns about the long-term effects have been raised [7-11]. It has been indicated that for 30-60% of patients participating in pain management programs, the treatment gain is not maintained long-term (at 1- to 5-year follow-ups) [8,10]. The need for strategies to maintain treatment effects by supporting self-management following treatment has received little attention in the research field [8].

An increasing number of studies on Internet-based interventions, many based on CBT (iCBT), indicate their efficacy in supporting use of constructive self-management strategies in individuals living with chronic illness [12-15]. The results of research on iCBT for individuals with chronic pain are not entirely consistent, but in a recent systematic review, it was concluded that Internet-based interventions seem promising [16]. At least three recent randomized trials, not included in this review, support this conclusion. A randomized trial of an intervention consisting of a website with self-management information based on CBT and no therapist contact was found to reduce pain and increase physical functioning in individuals with fibromyalgia, compared with a control group receiving standard care alone, at a 6-month follow-up [17]. Another study, testing the effect of a no-therapist contact online intervention found positive effects on pain, catastrophizing, and disability for the intervention group [18]. In the third study, persons with persistent symptoms after multidisciplinary pain management rehabilitation received 8 weeks of guided iCBT. There was a medium between-group effect between the intervention group and the active control group, but a small within-group effect on catastrophizing after the intervention, and the improvements were maintained after 6 months [19].

To date, research on the effects of iCBT for persons with pain beyond 6-month follow-up is limited. Additionally, only a few studies on iCBT have investigated the effect of an intervention aiming to support self-management following participation in a traditional pain management program [19,20].

Most iCBT interventions for chronic pain are based on weekly modules with self-help material and involve weekly written communication with a therapist [16,19]. A few studies have investigated a different approach to iCBT with daily communication with a therapist over a few weeks, using a personal digital assistant (PDA) or smartphone [20-24]. The use of a smartphone instead of a desktop or laptop computer gives the participant the flexibility to register and receive information in different situations of daily life. In these studies, diaries with questions aiming to support awareness of disability-related thoughts (eg, catastrophizing) and feelings have been made available to patients on a Web-enabled mobile phone or a smartphone. Instead of weekly feedback from a therapist, the participants receive a daily written message personalized according to the recently registered information. Two randomized trials provide evidence for positive short-term effects (3-month/5-month follow-up) [20,24].

In our randomized controlled study, 135 women with CWP completing a 4-week inpatient rehabilitation program were included [20]. A large effect on catastrophizing was found between the groups for the completers after receiving personalized feedback via a smartphone for 4-weeks. At 5-month follow-up, the effects remained moderate for catastrophizing, acceptance of pain, and functioning and symptom level [20]. The objective of the present paper is to report long-term results of the previously published trial on the smartphone intervention, ie, involving the same study with the same sample [20]. It was hypothesized that the intervention group would report less catastrophizing, better functioning, increased acceptance of pain, and success in values-based living than the control group at 11-month follow-up.

## Methods

### Study Design

**Figure 1:**
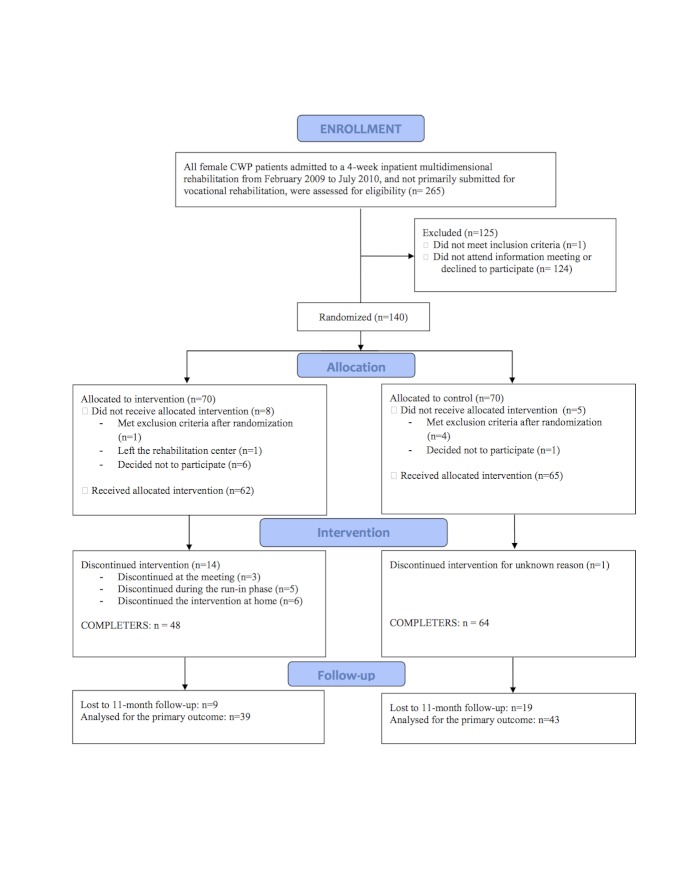
Study flow chart.

The overall study design is shown in Figure 1. The design is a parallel-group randomized controlled trial. Further details of the study can be found in our earlier publication from this trial [20].

All participants attended a 4-week inpatient multidimensional rehabilitation program for chronic pain. The program included education in pain mechanisms and CBT-based pain management (approximately 20 hours), various forms of aerobic exercise, stretching, relaxation, individual myofascial pain treatment, and medication was administered as needed (see [25] for details of the program). In the fourth week of the program, participants were randomly assigned to one of the two study groups. A detailed description of the recruitment procedure is given in the previous report [20].

The intervention group received a smartphone intervention for 4 weeks after completing the inpatient rehabilitation. Both groups were given access to an informational website with self-help pain-management material. Self-reported assessments on paper were gathered at five time-points: before (T1) and after (T2) the inpatient program, 4 weeks after discharge (T3) when the intervention group had completed their smartphone intervention, and 5 (T4) and 11 months (T5) after the smartphone intervention period (ie, 12 months after discharge from the inpatient rehabilitation program). The first two questionnaires were received and completed at the rehabilitation center and the others were completed at home and returned by mail. In this paper, results of the first two assessments (T1 and T2) and the last (11-month follow-up, T5) are reported. The customary self-report administration mode at the rehabilitation center was a paper-and-pencil format and was therefore used in this study.

#### Participants

Participants were recruited consecutively from Jeløy Kurbad Rehabilitation Centre in Moss, Norway. Patients were referred to the center by their general practitioner, a medical specialist, or from a hospital. The inclusion criteria for the study were: female, 18 years or older, participating in the inpatient program for persons with chronic pain, having suffered from CWP for more than 6 months (with or without a diagnosis of fibromyalgia), not participating in another research project at the rehabilitation centre, being able to use the smartphone, and not being diagnosed with a profound psychiatric disorder.

#### Ethical Aspects

The study was approved by the Regional Ethics Committee in South-East Norway and by the Norwegian Social Science Services. All participants signed an informed consent form. The study is registered at ClinicalTrial.gov (NCT01236209).

#### Assessment Measures

The Pain Catastrophizing Scale (PCS [26]) was used as the primary outcome variable of the study. It is a 13-item questionnaire with questions on helplessness, magnification, and rumination. Patients rate items on a scale from 0 (not at all) to 4 (all the time). The total score range for the PCS is 0-52, with higher scores reflecting higher degrees of catastrophizing. In our sample, the internal consistency was high on all assessments (Cronbach alpha = .89-.94). Catastrophizing is among the psychological constructs that can play an important role in the development and maintenance of chronic pain [27,28]. Catastrophizing has consistently been found to be associated with distress and disability [28]. The Chronic Pain Acceptance Questionnaire (CPAQ ) was used. It is scored on a 7-point Likert scale from 0 (never true) to 6 (always true) to give the total score (0-120). Higher scores reflect higher acceptance of pain and higher activities engagement. In our study, the Cronbach alpha coefficients were .81-.92. Emotional distress was measured with the questions from the 12-item General Health Questionnaire (GHQ) [29] with modified response alternatives. Bimodal scoring method was used (symptom present more than usual = 1, symptom present less than or as usual = 0). Total score range is 0 to 12; indicating the number of symptoms present more than usual during the last 2 weeks. In the current study, the Cronbach alpha coefficients were .72-.88. The Chronic Pain Values Inventory (CPVI) is a 12-item measure of importance and success in living according to one’s own values in six domains (family, intimate relationships, friendship, work, health, and personal growth) [30]. Each item is rated on a scale from 0 to 5, with higher numbers indicating more importance or success. The mean success rating was used as a measure of values-based action (score range: 0-5). In the present study, the Cronbach alpha coefficients for the success scale were .75-.88. The original (1991) version of fibromyalgia impact questionnaire (FIQ) was used to measure the impact of fibromyalgia on functioning and symptom levels the last week. The score range is 0 to 100; a higher score indicates greater impairment [31]. The Cronbach alpha coefficients were .78-.87 (two questions related to work were excluded because of high missing rates). Short-form health survey (SF-8) was also used to measure functioning. Summary measure scales for mental health component and physical component were obtained by using SF-8 Scoring Software 4.5 [32]. The standardized scores have a mean of 50 and a SD of 10. Higher scores indicate better functioning. The Cronbach alpha for the mental component were .65-.74 and .79-.85 for the physical component.

The current levels (last couple of days) of pain, fatigue, sleep disturbance, and depression were assessed on visual analogue scales (VAS) from 0 (no pain/fatigue/sleep disturbance/depression) to 100 (worst imaginable). One question on subjective global improvement was included: “How do you feel now compared to before you attended the inpatient program?”

### Treatment Procedures

#### Smartphone Intervention: Diaries and Daily Situational Feedback (Intervention Group Only)

The main theoretical framework was based on the cognitive behavioral fear-avoidance model [27] and CBT, and comprised, more specifically, elements from the acceptance and commitment therapy (ACT) [33,34]. ACT has been found to reduce catastrophizing and disability in chronic pain patients [35-37]. The aim was to support continued use of the self-management strategies learned at the rehabilitation center (eg, exercise and stretching) and to promote improved daily functioning and values-based living. In the rehabilitation center, a traditional CBT approach was used, not ACT. Therefore, ACT elements such as mindfulness exercises were added as new components. The smartphone intervention had the following four components.

##### Face-to-Face Session

The intervention started with an approximately 1-hour individual session between a nurse and the participant. The session took place in the last week before discharge. The participants received information (name and qualifications) about their therapist for the intervention, which, in some cases was the nurse at the meeting. The nurse attending the face-to-face session summarized the meeting and passed this background information to the relevant therapist. For the duration of the study, the participant was lent a smartphone and could call a member of the research group (OBK, HE) for technical support.

##### Web-Based Diaries

**Figure 2:**
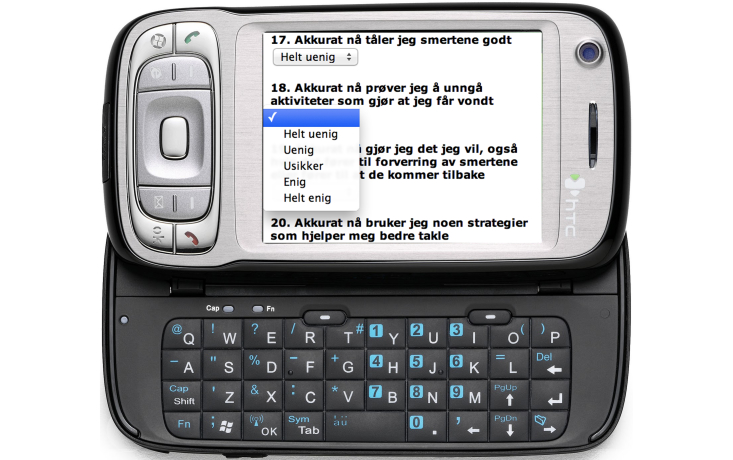
The smartphone's screen showing a diary in Norwegian.

**Figure 3:**
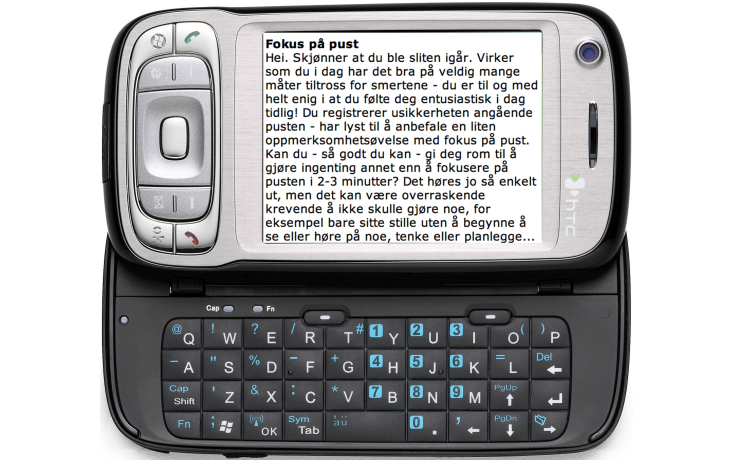
The smartphone's screen showing feedback in Norwegian.

The participant was asked to complete three diary entries per day using the smartphone. Examples of the smartphone’s screen display are shown in Figures 2 and 3. The diaries included 16-24 questions about the current level and interference of pain, feelings, and thoughts related to avoidance, catastrophizing, and acceptance. They also included questions about planned and previous use of self-management activities learned at the rehabilitation center and daily values-based and practical activities. Lists of self-management activities (eg, mild exercise, stretching, resting, aerobic exercise, pleasurable activity) were provided as a reminder. The questions were chosen to support awareness and reflection of experience relevant to self-management. Participants answered most questions by choosing predefined alternatives or using scales. The diaries included a comment field giving participants the opportunity to write a short personal message to the therapist.

At the time scheduled for diary completion, a short message service (SMS) message with a link to a secure website, where the diary could be opened and questions answered and posted, was received by the participant. The participants completed the first diary entry during the face-to-face session and continued during the last week before discharge with the goal of getting used to the diaries before discharge (a run-in period). After discharge, the diaries were completed every day for 4 weeks.

##### Written Personalized Feedback

For 4 weeks after discharge, excluding weekends, participants received daily written feedback from a therapist. The feedback was empathic and personalized according to each participant’s situation as reported in the diary. It included repetition of content reported in the diaries, positive reinforcement, reminders of self-management information given at the rehabilitation center, ACT exercises, and reflective questions. The aim was to encourage nonjudgmental awareness of cognitions, feelings, and emotions and to stimulate mindfulness and willingness to engage in meaningful activities despite pain or other discouraging intrusions, eg, to reduce the impact of catastrophizing on self-management behavior. The instructions for the exercises were written directly in the feedback or the participant was referred to exercises available on the smartphone and/or the website (see below). The feedback was also personalized according to the summary of personal information given at the face-to-face session (eg, family situation and health-related goals) and results on self-reported discrepancy between values and values-based living assessed with the CPVI at the end of the rehabilitation program. The feedback was usually available for the participant within 90 minutes after they had completed the second diary of the day. If this diary was not submitted, feedback based on information from the latest submitted diary was sent. There was no limitation on the length of the feedback, which ranged from a few sentences to a few paragraphs.

The feedback was written by any of 3 of the authors (OBK, TLS, and HE); all with a background in health care sciences (nursing and/or psychology).

##### Audio Files With Guided Mindfulness Exercises

A few audio files with short mindfulness exercises guided by the authors were available on the smartphones.

#### Informational Website With Self-Help Pain Management Material (All Participants; Control Group Received Only This Intervention)

All participants received access to a static website with information on self-management strategies for people with chronic pain. The website also included a few written ACT exercises and audio files with mindfulness exercises (as described above). See Multimedia Appendix 1 for a screenshot from the website. No specific instruction about frequency of use was given.

### Statistics

To investigate differences in demographic variables and baseline characteristics, independent sample *t*-tests, nonparametric tests, and Chi-square tests were used. Paired *t*-tests were used to compare 11-month follow-up (T5) results to the baseline for the inpatient program (T1) and the smartphone intervention (T2). Independent *t*-tests or nonparametric tests (Mann-Whitney) were used to compare outcomes between groups at T5. The Cohen’s *d* effect sizes (ES) were calculated using the difference between the groups’ means divided by the mean of the standard deviation of both groups. If one or two items were missing on the GHQ, they were scored as present less than usual or as usual (= 0). If another instrument included one or two missing items, the items were replaced with the mean of other items from the participant’s instrument. If two response alternatives were marked, the healthier option was chosen. Total score was not computed if more than two items were missing, and the case was categorized as missing a total score for the instrument. The number of participants included in each analysis is provided. In the intention-to-treat analysis (ITT), the results of complete case analysis for the primary outcome is reported. In addition, two methods for replacing missing variables for the primary outcome at endpoint (T5) were applied: last observation carried forward (LOCF) and multiple imputations (MI). In the MI analysis, 50 imputations were made. The following clinically significant variables were included in the MI regression model: age, SF-8 physical component, and VAS for pain, sleep, fatigue, and depression at admission to the rehabilitation center. Six of the participants who withdrew from the smartphone intervention contributed questionnaires at the 11-month follow-up (T5). The ITT analyses included all participants (n=135) except those who met the exclusion criteria after randomization. In the analyses of secondary outcomes, only those who completed the interventions were included (n=112). IBM SPSS Statistics (versions 19 and 20) was used. A significance level of *P*=.05 was chosen, and a tendency toward difference was defined as *P*<.1. Effect sizes were categorized as small (<.5), medium (.5-.8), and large (>.8) in accordance with Cohen [38].

## Results

### Participants

265 women who were eligible for the study during the study period were invited to informational meetings about the project. Of these, 124 did not attend a meeting or declined to participate, and 1 did not meet the inclusion criteria. 140 were randomized to one of the two study arms (Figure 1). 5 subjects met the exclusion criteria after randomization (they were originally submitted for vocational rehabilitation and thus included in another research project), and 8 discontinued participation before receiving the allocated intervention. In the intervention group, 14 patients did not complete the intervention. Demographic data and baseline characteristics of the sample by groups are given in Table 1. All participants had CWP, and the majority was diagnosed with fibromyalgia. Despite randomization, the groups differed in mean pain level (*P*=.02) and physical functioning measured by SF-8 (*P*=.03) at admission to the rehabilitation center.

### Primary Outcome: Catastrophizing

Descriptive results for catastrophizing are shown in Table 2 and follow-up differences in Table 3.

### Between-Group Effects

At the 11-month follow-up (T5), there was no difference between the groups on the measure of catastrophizing (PCS); neither according to the ITT-analysis (LOCF *P*=.22 and MI *P*=.31) nor the per protocol analysis (complete case analysis, *P*=.18 and analysis with MI .23). When all intended-to-treat were analyzed (LOCF), catastrophizing seemed to improve more during the follow-up period (T2-T5) in the intervention group (M=-2.36, SD 8.41) than the control group (M=.40, SD 7.20), *P*=.045, using *t*-test of change scores.

### Within-Group Effects

There were small positive within-group effects for the intervention group between T2 and T5 on catastrophizing by ITT (LOCF) and per protocol analyses, *P*=.02 and *P*=.04, respectively. In the analysis, where missing variables were imputed, there was a tendency towards a small positive within-group effect during this period (T2-T5). There were improvements regarding to catastrophizing in the T1 to T5 period for both the intervention and control groups, *P*<.001. Between 5-month follow-up (T4) and 11-month follow-up (T5), paired samples *t*-tests did not show changes in the intervention group (M=.06, SD 5.05, n=31), *P*=.94 for catastrophizing. However, in this period (T4-T5), there was a reduction in catastrophizing in the control group (M=-3.25, SD 7.09, n=34), *P*=.01.

### Secondary Outcomes: Functioning and Symptom Levels

Table 4 shows descriptive statistics for the secondary outcomes at admission to the rehabilitation center, at discharge, and at the 11-month follow-up. In the per protocol analysis, no significant group differences were detected at discharge from the rehabilitation center on any of the outcome variables (all *P*s>.05; GHQ, and depression (VAS), *P*=.08, see Table 4).

### Between-Group Effects

No between-group differences were found at the 11-month follow-up; *P*-values ranged from .13 (SF-8, physical) and .17 (CPAQ) to .98 (sleep disturbance).

### Within-Group Effects

Table 5 shows within-group changes for the secondary outcomes. When comparing the smartphone intervention’s baseline data (T2) to the 11-month follow-up (T5), there was a moderate positive effect on acceptance (CPAQ) in the intervention group, but not in the control group. There was a small negative effect on functioning and symptom levels measured by the FIQ in the control group, but not in the intervention group. For the physical component of SF-8, there was a small negative effect for the intervention group (*P*=.046), but not the control group. For GHQ, there was a large negative effect for emotional distress but only in the control group. No significant changes were detected for the mental component of SF-8. There was a tendency towards improvement in values-based living in the intervention group but not in the control group. There was a moderate negative effect on fatigue (VAS), and a tendency towards a small negative effect on sleep (VAS) and pain (VAS) in the control group. No changes were detected on these symptoms in the intervention group.

When comparing baseline data for the inpatient program (T1) to the follow-up data (T5), improvement in acceptance, mental health measured by SF8, and values-based living was found in both groups (see Table 5). Reduction in disease impact (measured by FIQ) was found for the intervention group only (Cohen’s *d*=.42, *P*=.03). There was a significant reduction in pain level in the intervention group between T1 and T5, mostly due to changes during the inpatient program (see Table 4). There was a trend towards improvement on fatigue and depression (VAS scales) in the intervention group only, between T1 and T5. When both groups were analyzed together, with all intended-to-treat included (complete case analysis), there was a reduction in pain level (M=5.68, SD 24.66, n=89) between admission to the inpatient program (T1) and the long-term follow-up (T5), *P*=.03.

When the 5-month follow-up results (T4) were compared to the 11-month follow-up (T5), no changes were found for acceptance, pain level, functioning, and symptom level (measured by FIQ), sleep disturbance, fatigue, and mental health (all *P* values > 0.10). During this period (T4-T5), the control group showed improvement in values-based living (M=.25, SD .70, n=33, *P*=.046), whereas the intervention group did not. The control group also showed improvement in depression (measured by a VAS, M=8.29, SD 20.13, n=35, *P*=.02), whereas no significant change was found in the intervention group during this (T4-T5) period. Reduction in physical functioning (measured by SF8) was found in the intervention group (M=3.45, SD 7.76, n=31, *P*=.02) and a trend towards improvement in the control group (M=2.30, SD 7.10, n=34, *P*=.07).

Of the completers, 47.4% (n=18) in the intervention group and 40.0% (n=18) in the control group reported feeling better now than before the inpatient program. 13.1% (n=5) in the intervention group and 11.1% (n=5) in the control group reported feeling worse now compared to before the inpatient program. No change was reported by 39.5% (n=15) in the intervention group and by 48.9% (n=22) in the control group.

### Withdrawal From Participation

Of the 135 participants (of 140 randomized) that met the inclusion criteria, 112 completed the study period (Figure 1). 21 withdrew from the intervention group (30.4%) and 2 withdrew from the control group (3.0%). There was a trend toward the completers being younger (M=43.33, SD 11.18) than the ones who withdrew (M=48.43, SD 10.06), *P*=.07. Additionally, there was a trend towards a higher level of depression (measured by a single VAS) in the group who withdrew (M=43.62, SD 28.57) compared to the completers (M=31.81, 28.92), at admission to the inpatient program, *P*=.06.

### Response Rates to 11-month Questionnaires in Intervention Group and Control Group

The response rate for all included participants (n=135) was 66.7% at 11-month follow-up (T5) (n=45 in the intervention group and n=45 in the control group). When only completers (n=112) were included, the response rate at T5 was 81.3% (n=39) in the intervention group and 70.3% (n=45) in the control group. Among the completers, those who returned the questionnaire at T5 had better physical functioning (M=34.60, SD 7.53), at admission to the inpatient program measured by SF8, compared to those who did not return them (M=31.19, SD 6.93), *P*=.01. Those who returned the questionnaires at T5 reported also less disease impact (M=56.45, SD 16.82) on FIQ at admission to the inpatient program compared to those who did not return them (M=64.22, SD 14.59), *P*=.03. Those who returned the T5 assessments reported more success in living according to values (M=2.13, SD .81) than those who did not return them (M=1.74, SD .85), *P*=.04. There was also a tendency towards more severe depression (measured by a VAS scale) at both admission and discharge among those who did not return questionnaires at T5 compared to those who returned them, *P*=.08-.09.

**Table 1:** Characteristics at admission to the inpatient rehabilitation center.

Characteristic		Smartphone intervention (n=69)^a^	Control (n=66)^a^
Age, mean (SD), n		44.59 (11.13), 69	43.80 (11.20), 65
**Marital status**			
	Married or cohabiting	60.9% (n=42)	68.2% (n=45)
	Divorced	13.0% (n=9)	9.1% (n=6)
	Single	18.8% (n=13)	15.2% (n=10)
	Widow	5.8% (n=4)	3.0% (n=2)
	Unknown	1.4% (n=1)	4.5% (n=3)
**Years of education**			
	≤ 10 years (elementary)	18.8% (n=13)	12.1% (n=8)
	11-13 years (high school)	27.5% (n=19)	45.5% (n=30)
	>13 years (College/University)	43.5% (n=30)	34.8% (n=23)
	Unknown	10.1% (n=7)	7.6% (n=5)
**Employment status**			
	Working/studying	21.7% (n=15)	12.1% (n=8)
	Unemployed	4.3% (n=3)	1.5% (n=1)
	On sick leave	39.1% (n=27)	51.5% (n=34)
	On disability pension	17.4% (n=12)	19.7% (n=13)
	Part time working/studying and part time sick leave	11.6% (n=8)	7.6% (n=5)
	Other combination of the above	5.8% (n=4)	6.1% (n=4)
	Unknown	0%	1.5% (n=1)
Diagnosed with fibromyalgia (valid %)		80.9% (n=55)	84.4% (n=54)
Duration of symptoms (years), mean (SD), n		13.11 (8.78)	15.47 (12.09)
PCS^b^, mean (SD), n		21.24 (10.33), 63	20.80 (9.45), 62
CPAQ^b^, mean (SD), n		56.48 (15.02), 58	53.87 (13.81), 57
FIQ^b^, mean (SD), n		58.75 (16.39), 69	58.58 (16.04), 66
SF-8, physical^b^; mean (SD), n		31.91 (7.57), 65	34.75 (7.35), 62
SF-8, mental^b^, mean (SD), n		39.33 (10.49), 65	39.34 (9.61), 62
GHQ-12^b^, mean (SD), n		3.32 (3.38), 62	3.02 (3.38), 61
CPVI^b^, mean (SD), n		2.07 (0.95), 64	2.01 (0.73), 61
**VAS ^b^ recordings of current level of (last couple of days):**			
	Pain, mean (SD), n	67.08 (17.47), 69	57.85 (21.60), 66
	Fatigue, mean (SD), n	67.40 (23.73), 69	64.72 (21.02), 66
	Sleep disturbance, mean (SD), n	57.24 (26.22), 68	55.16 (23.38), 66
	Depression, mean (SD), n	34.73 (29.15), 68	32.93 (29.26), 65

^a^ Patients meeting exclusion criteria after randomization were not included in this analysis.

^b^ VAS, visual analogue scale (0-100^c^); PCS, Pain Catastrophizing Scale (score range 0-52^c^); CPAQ, Chronic Pain Acceptance Questionnaire (score range 0^c^-120); FIQ, Fibromyalgia Impact Questionnaire (0-100^c^); SF-8 (0^c^-100), Short Form; GHQ-12, questions from the General Health Questionnaire (score range 0-12^c^); CPVI, Chronic Pain Values Inventory (success score, range 0^c^-6).

^c^ Values that indicate maximum symptom scores/least health.

**Table 2:** Means and standard deviations for the primary outcome at admission to the inpatient rehabilitation (T1), at discharge (T2), and 11 months (T5) after the intervention period.

Primary outcome measure, Pain Catastrophizing Scale	Group	T1^a ^ Mean (SD), n	T2^a, b ^ Mean (SD), n	T5^a ^ Mean (SD), n
**ITT (complete case analysis)**				
	Intervention	21.24 (10.33), 63	15.12 (9.61), 63	11.50 (8.68), 44
	Control	20.80 (9.45), 62	15.41 (9.62), 59	14.73 (9.95), 43
**ITT(LOCF)**				
	Intervention	21.24 (10.33), 63	16.06 (10.37), 68	13.72 (10.02), 69
	Control	20.80 (9.45), 62	15.33 (9.31), 65	15.57 (10.40), 66
**ITT (MI)**				
	Intervention			12.80, 69
	Control			14.74, 66
**Per protocol (complete case analysis)**				
	Intervention	20.56 (10.08), 43	14.61 (8.93), 45	11.92 (8.97), 39
	Control	20.78 (9.59), 60	15.46 (9.76), 57	14.73 (9.95), 43
**Per protocol (MI)**				
	Intervention			12.25, 48
	Control			14.66, 64
**% (valid) with PCS score >24**				
	Intervention	30.2%, 13	15.6%, 7	12.8%, 5
	Control	33.3%, 20	17.5%, 10	18.6%, 8

^a^ T1, at admission to the inpatient program; T2, at discharge from the inpatient program; T5, 11-month follow-up

^b^ No differences between groups at T2 (all *P* values >.05).

**Table 3:** Within-group differences and effect sizes (ES) for the primary outcome.

Primary outcome, PCS		Mean difference T2-T5^a^ (n)	95% CI T2-T5	ES for T2-T5	*P*-value^b^	Mean difference T1-T5^a^ (n)	95% CI T1-T5	ES for T1-T5	*P*-value^b^
**ITT (complete case analysis)**									
	Intervention	-2.60 (8.72), 42	-5.32, .11	.29	.06	-7.36 (7.87), 39	-9.91, -4.81	.80	< .001
	Control	-.21 (7.32), 40	-2.55, 2.13	.02	.86	-5.30 (7.30), 41	-7.60, -2.99	.56	< .001
**ITT (LOCF)**									
	Intervention	-2.36 (8.41), 68	-4.39, -.32	.23	.02	-7.57 (8.23), 63	-9.64, -5.50	.76	< .001
	Control	.40 (7.20), 65	-1.39, 2.18	-.04	.66	-4.65 (7.43), 62	-6.54, -2.76	.47	< .001
**ITT (MI)**									
	Intervention	-2.61, 63	-5.33, .10		.06	-8.22, 63	-10.86, -5.57		< .001
	Control	-.15, 59	-2.75, 2.46		.91	-5.79, 62	-8.12, -3.46		< .001
**Per protocol (complete case analysis)**									
	Intervention	-3.04 (8.74), 37	-5.95, -.12	0.33	.04	-7.23 (8.01), 34	-10.03, -4.44	.77	< .001
	Control	-.21 (7.32), 40	-2.55, 2.13	0.02	.86	-5.30 (7.30), 41	-7.60, -2.99	.56	< .001
**Per protocol (MI)**									
	Intervention	-2.45, 45	-5.29, .38		.09	-7.93, 43	-10.72, -5.14		< .001
	Control	-.27, 57	-2.84, 2.30		.84	-5.85,60	-8.15, -3.54		< .001

^a^ T1, at admission to the inpatient program; T2, at discharge from the inpatient program; T5, 11-month follow-up.

^b^ *P* values for paired samples *t*-tests.

**Table 4:** Means and standard deviations for the secondary outcomes at admission to the inpatient rehabilitation (T1), at discharge (T2), and 11 months (T5) after the intervention period, for the completers.

Secondary outcome measures	Group	T1^a ^ Mean (SD), n	T2^a,b ^ Mean (SD), n	T5^a ^ Mean (SD), n
**CPAQ**				
	Intervention	56.45 (15.22), 40	62.00 (13.62), 44	71.62 (14.11), 39
	Control	53.94 (13.92), 56	62.21 (10.15), 57	67.05 (15.18), 42
**FIQ**				
	Intervention	58.46 (17.26), 48	46.38 (16.92), 47	49.24 (21.34), 38
	Control	58.35 (16.18), 64	49.10 (17.32), 62	53.75 (17.73), 45
**SF-8, physical**				
	Intervention	32.12 (7.74), 45	36.68 (8.42), 40	34.38 (9.88), 39
	Control	34.98 (7.13), 60	35.86 (8.24), 49	37.35 (7.65), 44
**SF-8, mental**				
	Intervention	39.50 (10.67), 45	45.70 (8.06), 40	45.50 (10.70), 39
	Control	39.09 (9.61), 60	44.83 (9.69), 49	43.87 (9.09), 43
**GHQ-12**				
	Intervention	3.19 (3.21), 43	1.20 (2.02), 45	1.95 (2.64), 38
	Control	2.97 (3.43), 59	0.63 (1.01), 57	2.20 (2.82), 44
**CPVI**				
	Intervention	2.05 (0.95), 44	2.47 (0.91), 46	2.78 (1.00), 39
	Control	2.02 (0.74), 59	2.52 (0.68), 54	2.50 (0.77), 43
**Pain, VAS**				
	Intervention	66.59 (17.58), 48	53.07 (22.20), 47	56.28 (28.24), 38
	Control	57.32 (21.56), 64	52.99 (21.27), 61	55.85 (22.73), 45
**Fatigue, VAS**				
	Intervention	69.29 (23.98), 48	51.38 (27.75), 47	60.79 (28.56), 38
	Control	64.08 (21.01), 64	50.10 (24.28), 61	61.63 (23.63), 45
**Sleep disturbance, VAS **				
	Intervention	54.77 (26.99), 47	43.97 (25.77), 47	51.68 (30.45), 38
	Control	54.59 (23.31), 64	48.12 (24.57), 62	53.08 (25.95), 45
**Depression, VAS**				
	Intervention	30.68 (28.71), 47	19.84 (24.08), 47	22.82 (25.89), 38
	Control	32.65 (29.29), 63	27.36 (28.51), 61	28.93 (27.71),45

^a^ T1, at admission to the inpatient program; T2, at discharge from the inpatient program; T5, 11-month follow-up.

^b^ No differences between groups at T2 (all P values >.05; GHQ and depression (VAS), *P*=.08).

**Table 5:** Mean differences for the secondary outcomes within-groups, confidence intervals (CI) and effect sizes (ES) for the completers.

Secondary outcome measures		Mean difference T2-T5 (n)^a^	95% CI T2-T5^a^	ES for T2-T5^a^	*P*-value^b^	Mean difference T1-T5 (n)^a^	95% CI T1-T5^a^	ES^a^ for T1-T5	*P*-value^b^
**CPAQ**									
	Intervention	10.20 (9.57), 35	6.91, 13.49	.73	<.001	14.12 (12.46), 33	9.70, 18.54	.95	< .001
	Control	2.15 (10.77), 39	-1.34, 5.64	.17	.22	8.45 (14.00), 38	3.85, 13.05	.62	.001
**FIQ**									
	Intervention	1.40 (21.15), 37	-5.65, 8.45	-.08	.69	-8.15 (21.95), 38	-15.36, -.93	.42	.03
	Control	7.05 (17.24), 44	1.81, 12.29	-.40	.01	-2.27 (16.23), 45	-7.15, 2.60	.13	.35
**SF-8, physical**									
	Intervention	-3.20 (8.84), 33	-6.33, -.06	-.36	.046	1.11 (8.99), 36	-1.93, 4.15	.12	.46
	Control	.54 (8.18), 34	-2.32, 3.39	.06	.71	1.01 (6.99), 42	-1.17, 3.19	.14	.36
**SF-8, mental**									
	Intervention	.39 (10.09), 33	-3.19, 3.97	.04	.82	6.45 (11.71), 36	2.49, 10.42	.58	.002
	Control	-1.61 (11.53), 33	-5.70, 2.48	-.17	.43	3.10 (6.99), 42	.93, 5.28	.36	.006
**GHQ-12**									
	Intervention	.69 (3.06), 35	-.36, 1.74	-.28	.19	-1.12 (4.07), 34	-2.54, .30	.36	.12
	Control	1.68 (2.94), 40	.74, 2.61	-.85	.001	-.61, 3.39, 41	-1.68, .46	.20	.26
**CPVI**									
	Intervention	.29 (.90), 37	-.01, .59	.30	.06	.56 (.72), 35	.32, .81	.59	< .001
	Control	-.11 (.88), 36	-.41, .19	-.16	.46	.40 (.61), 41	.21, .59	.54	< .001
**Pain, VAS**									
	Intervention	1.43 (32.58), 37	-9.44, 12.29	-.06	.79	-9.86 (25.54), 38	-18.25, -1.46	.43	.02
	Control	5.53 (20.73), 43	-.85, 11.91	-.25	.09	-1.82 (23.65), 45	-8.92, 5.28	.08	.61
**Fatigue, VAS**									
	Intervention	8.71 (33.56), 37	-2.48, 19.90	-.32	.12	-8.94 (31.93), 38	-19.43, 1.56	.36	.09
	Control	14.96 (24.50), 43	7.42, 22.49	-.61	<.001	-.93 (22.65), 45	-7.73, 5.87	.04	.78
**Sleep disturbance, VAS**									
	Intervention	5.55 (33.26), 37	-5.54, 16.64	-.20	.32	-2.29 (33.46), 37	-13.45, 8.86	.08	.68
	Control	7.43 (27.09), 44	-.81, 15.66	-.28	.08	.45 (30.11), 45	-8.59, 9.50	-.02	.92
**Depression, VAS**									
	Intervention	1.11 (28.71), 37	-8.46, 10.68	-.05	.82	-8.13 (27.78), 37	-17.40,1.13	.29	.08
	Control	7.27 (27.43), 43	-1.18, 15.71	-.26	.09	.99 (23.13), 45	-5.96, 7.94	-.04	.78

^a^T1, at admission to the inpatient program; T2, at discharge from the inpatient program; T5, 11-month follow-up.

^b^ *P* values for paired samples *t*-tests.

## Discussion

The results of the study are ambiguous. On one hand, there were no significant differences in mean values on any variables between the groups at 11-month follow-up. Thus, the favorable effects previously reported on catastrophizing, acceptance, functioning, and symptom levels at 5-month follow-up were not evident at long-term follow-up. However, there was significantly more improvement in catastrophizing scores during the follow-up period in the intervention group compared to the control group. Moreover, the within-group analyses, comparing the baseline for the smartphone intervention to the 11-month data, revealed changes in the desired direction in catastrophizing and acceptance in the intervention group but not within the control group. Also, increase in disease impact, emotional distress, and fatigue were seen in the control group but not within the intervention group. Additionally, effects on most variables were maintained in the intervention group from the 5-month follow-up to the 11-month follow-up. Unexpectedly, between the two follow-ups, the control group reported some improvement in several variables (catastrophizing, values-based living, and depression) whereas the intervention group did not. We have no data to support an explanation for this improvement. One could speculate that it takes time for changes in thoughts, behavior, and priorities promoted by the multidimensional inpatient rehabilitation program to settle and cause positive effects. The controls did not get the smartphone intervention that could promote these changes early after discharge, and thus the changes may have been achieved at an earlier stage in the intervention group. We do not have exact login information for visits to the website. As mentioned in our previous paper, most participants in the control group (26 of the 38 who reported this information) visited it rarely (2 times or less). The impression of the administrator of the website (HE) was that it was seldom accessed. Based on the limited use of the website, it is not assumed to have caused any changes seen in the control group. The spontaneous improvement in the control group, large variations within variables, relatively few participants, and small effect sizes may explain the lack of significant differences between the groups. It is important to acknowledge that the effects of the inpatient program were sustained at the long-term follow-up in the control group for many of the outcome variables, ie, catastrophizing, acceptance, mental health, and values-based living. Improvement in those variables indicates that the participants cope better with their situation.

Some of the limitations regarding the generalizability of the study have been discussed in the previous report, eg, the difference in the completers groups versus those withdrawing [20]. Again, at this follow-up we have the limitations of a response rate below 70%. Those not returning the follow-up questionnaires reported generally more symptoms at admission to the inpatient program than those who returned them, thus having the possibility to influence the results. Multiple imputations have been recommended to improve the validity of results in trials with incomplete datasets [39]. In the ITT analysis, the level of catastrophizing in the control group at endpoint (T5) was almost the same for the complete case analysis (mean 14.73, n=43) and the MI analysis (mean 14.74, n=66). In the intervention group, the catastrophizing level was somewhat higher with MI (mean 12.80, n=69) compared to the complete case analysis (mean 11.50, n=44). This might partly be explained by higher baseline scores on two variables (pain and SF-8 physical component), which were included in the MI regression model. Importantly, in the per protocol analysis, the difference between the mean levels of catastrophizing with MI or without (complete case analysis) was small. This provides some support for the validity of our results of secondary outcomes, where results of complete case analysis is reported. However, in the within-group analysis, the difference between the intervention baseline (T2) and 11-month follow-up (T5) in the intervention group was significant applying complete case analysis (*P*=.04) but only borderline significant in the analysis with MI (*P*=.09), thus indicating that the results for complete case analysis should be interpreted with some caution.

The withdrawal rate of 30% indicates that this type of secondary intervention may not be found feasible by all. The withdrawal rate is similar to those reported in many iCBT, where an average dropout rate of 27% has been reported [40]. The patients who withdrew tended to score higher on depression and were older than the completers, which could have influenced their interest and capacity to participate. We do not have information on the reasons for withdrawal for all participants. However, many of those who withdrew before or during the run-in period reported that the combination of the smartphone intervention and participation in the inpatient program was stressful or expected to be stressful. Therefore, closer collaboration with the rehabilitation center and flexibility in start-up date of the smartphone intervention might contribute to reduction in withdrawal rates. At the 11-month follow-up, the subjective global improvement measure could have been improved by including a question to assess the participants’ evaluation of the smartphone intervention.

Medications, education, CBT, and physical exercises are among the recommended treatment options for individuals with CWP and fibromyalgia [7,9]. The short-term effects are well established, but concerns about the long-term effects have been raised. In a recent longitudinal study including 1555 patients with fibromyalgia receiving standard care, with a mean follow-up period of 4 years, no clinically meaningful improvement in overall symptom severity was found for the sample. Only about one fourth of the sample showed meaningful improvement, including 10% with substantial improvement in symptom severity [41]. The goals of most nonpharmacological treatment are to provide knowledge and teach self-management skills aiming to reduce symptoms or support constructive coping. Adherence to recommended self-management strategies after treatment seems important for long-term effect. The research literature on Internet-delivered interventions to support self-management in individuals with chronic pain is rapidly evolving, with studies on therapist-guided and unguided intervention, as well as on applications for smartphones [16,42,43]. The present smartphone intervention was primarily meant to support use of constructive coping skills and implementation of recommended lifestyle changes the first weeks after discharge from inpatient rehabilitation, in order to prevent the fading of positive effects from the given rehabilitation. The smartphone intervention introduced elements from ACT, including mindfulness, which had not been presented in the inpatient program. We do not know if this influenced the results. Nevertheless, we acknowledge that the smartphone intervention could have been more strongly integrated in the rehabilitation program, eg, including the same health care professionals. The feasibility and long-term efficacy of the intervention might possibly be improved by providing the diaries on the individuals’ own smartphones and by providing feedback on a more long-term basis. An 8-week, guided iCBT intervention following multidisciplinary treatment was found to reduce catastrophizing in a randomized trial with 72 persons with residual symptoms post treatment [19]. The intervention lasted twice as long as our intervention but included less therapist contact. The effects on catastrophizing were remained at 6-month follow-up, but more long-term effects are not reported [19]. A smartphone application based on ACT to support values-based living was found feasible in an exploratory study including 11 healthy volunteers [43]. Ways to tailor the diary content and provide tailored feedback should be investigated. Booster periods with therapist-feedback might be beneficial or a longer period with less frequent therapist contact, eg, once a week/month.

To conclude, the results of this randomized trial are ambiguous. No significant between-group effect was found on the study variables at 11-month follow-up. However, more improvement in catastrophizing scores was seen in the intervention group than the control group in the period between discharge from the inpatient program and the follow-up. Moreover, the within-group analyses, comparing the baseline for the smartphone intervention to the 11-month data indicated changes in the desired direction in catastrophizing and acceptance in the intervention group but not within the control group. Also, increases in disease impact, emotional distress, and fatigue were seen in the control group but not within the intervention group. This kind of smartphone intervention may therefore be suited for providing self-management support following inpatient pain management program. Research on strategies to provide feasible self-management support on a long-term basis for individuals with CWP and ways to enhance cost-effectiveness is needed.

## Multimedia Appendix 1

Screenshot from the website.

## Multimedia Appendix 2

CONSORT-EHEALTH Checklist V1.6.2 [44].

## Abbreviations

ACT: acceptance and commitment therapy
CBT: cognitive behavioral therapy
CPAQ: chronic pain acceptance questionnaire
CPVI: chronic pain values inventory
CWP: chronic widespread pain
ES: effect size
FIQ: fibromyalgia impact questionnaire
GHQ: general health wuestionnaire
iCBT: Internet-based cognitive behavioral therapy
ITT: intention-to-treat
LOCF: last observation carried forward
M: mean
MI: multiple imputations
PCS: pain catastrophizing scale
SF: short-form health survey
SMS: short message service
VAS: visual analog scale
