# Supplementary figures and images for: A Smartphone-Based Intervention With Diaries and Therapist Feedback to Reduce Catastrophizing and Increase Functioning in Women With Chronic Widespread Pain. Part 2: 11-month Follow-up Results of a Randomized Trial
Source: J Med Internet Res. 2013 Mar 28;15(3):e72. doi: 10.2196/jmir.2442 (PMC3636011; doi:10.2196/jmir.2442)

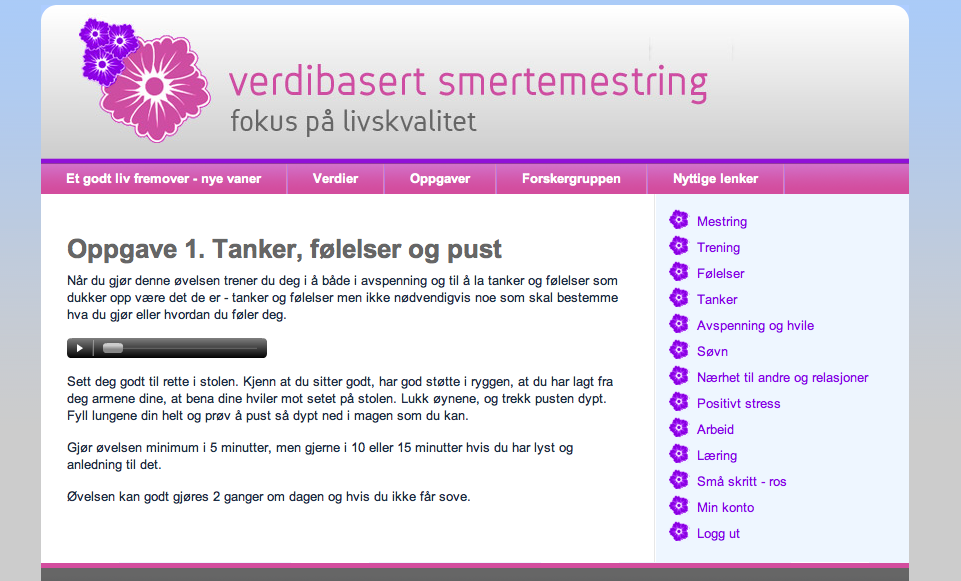

Supplement: Supplementary file 1 [file jmir_v15i3e72_app1.png]
