# Supplementary material for: A Smartphone-Based Intervention With Diaries and Therapist Feedback to Reduce Catastrophizing and Increase Functioning in Women With Chronic Widespread Pain. Part 2: 11-month Follow-up Results of a Randomized Trial
Source: J Med Internet Res. 2013 Mar 28;15(3):e72. doi: 10.2196/jmir.2442 (PMC3636011; doi:10.2196/jmir.2442)
Supplement: Supplementary file 2 [file jmir_v15i3e72_app2.pdf]

CONSORT-EHEALTH Checklist V1.6.2 Report

(based on CONSORT-EHEALTH V1.6), available at [http://tinyurl.com/consort-ehealth-v1-6].

2442

Date completed

10/22/2012 12:46:46

by

Masahiko Ando

Effectiveness of Web-Based Peer-Conscious
Health Support for Weight Loss:
A Randomized Controlled Trial

TITLE

1a-i) Identify the mode of delivery in the title

Web-Based Peer-Conscious Health Support for Weight Loss

1a-ii) Non-web-based components or important co-interventions in title

1a-iii) Primary condition or target group in the title

Weight Loss

ABSTRACT

1b-i) Key features/functionalities/components of the intervention and comparator in the METHODS section of the ABSTRACT

All participants in the WPS group could receive nutritional advice and calculate their nutritive intake from a photograph of a meal on their computer screen from the Internet sent to them by their dietitian, receive supervision from the registered dietitian, and view fellow participants' weight changes and lifestyle modifications. In the EMS group, a participant could receive one-to-one nutritional advice and calculate his/her nutritive intake from the photograph of a meal on computer screen sent by e-mail from his/her dietitian, without being able to view fellow participants' status.

1b-ii) Level of human involvement in the METHODS section of the ABSTRACT

1b-iii) Open vs. closed, web-based (self-assessment) vs. face-to-face assessments in the METHODS section of the ABSTRACT

This study was designed as an open prospective individual randomized controlled trial.

1b-iv) RESULTS section in abstract must contain use data

A total of 193 participants was randomly assigned to either the WPS group (97) or the EMS group (96). Ten from the WPS group and 8 from the EMS group dropped out during the study period, and the remaining 87 in the WPS group and 88 in the EMS group were followed-up completely. Weight loss was significantly greater in the WPS group than in the EMS group (-1.6 kg versus -0.7 kg, adjusted P=.038). However, there were few differences in waist circumference between the two groups. (-3.3 cm versus -3.0 cm, adjusted P=.708)

1b-v) CONCLUSIONS/DISCUSSION in abstract for negative trials

INTRODUCTION

2a-i) Problem and the type of system/solution

Obesity is one of the most common public health problems in the industrialized world as a cause of noncommunicable diseases such as ischemic heart disease and diabetes mellitus [1,2].
This study aimed to compare the weight loss between the WPS and the conventional e-mail health support (EMS).

2a-ii) Scientific background, rationale: What is known about the (type of) system

A study reported that people were more likely to gain weight when obese persons were around them [3]. In a similar way, behavior modification for weight loss might also 'transmit' to others if a person makes an effort to lose weight. In addition, the necessity to enhance the motivation for weight loss by group dynamics in nutritional counseling has been emphasized [4,5].
In nutritional counseling for weight loss, face-to-face support that takes into consideration the individual's background and personal characteristics is generally conducted by registered dietitians [6,7]. Recently, e-mails, which are mainly for one-to-one communication, have been frequently used for weight loss [8-13].

METHODS

3a) CONSORT: Description of trial design (such as parallel, factorial) including allocation ratio

This study aimed to compare the weight loss between the WPS and the conventional e-mail health support (EMS) . Our hypothesis is that weight loss would be greater in the WPS group than in the EMS group.

3b) CONSORT: Important changes to methods after trial commencement (such as eligibility criteria), with reasons

None.

3b-i) Bug fixes, Downtimes, Content Changes

4a) CONSORT: Eligibility criteria for participants

Men and women aged 35-65 years with a body mass index (BMI) of 25.0 or more from their latest health examination were eligible. Persons who agreed to participate in our study were invited to an initial face-to-face guidance interview. At this interview, those who had been receiving dietary and exercise therapies, or who could not access Internet or e-mail, or who had a current BMI less than 24.5, were excluded from our intervention.

4a-i) Computer / Internet literacy

4a-ii) Open vs. closed, web-based vs. face-to-face assessments:

This study was an open prospective individual randomized controlled trial.
This study aimed to compare the weight loss between the WPS and the conventional e-mail health support (EMS).
For this study, we recruited participants by mail, contacting clients of the Kyoto University Health Service, Japan, urging them to obtain nutritional counseling for weight loss.

4a-iii) Information giving during recruitment

4b) CONSORT: Settings and locations where the data were collected

The Kyoto University Health Service, Japan.

4b-i) Report if outcomes were (self-)assessed through online questionnaires

None.

4b-ii) Report how institutional affiliations are displayed

5) CONSORT: Describe the interventions for each group with sufficient details to allow replication, including how and when they were actually administered

5-i) Mention names, credential, affiliations of the developers, sponsors, and owners

5-ii) Describe the history/development process

5-iii) Revisions and updating

5-iv) Quality assurance methods

5-v) Ensure replicability by publishing the source code, and/or providing screenshots/screen-capture video, and/or providing flowcharts of the algorithms used

5-vi) Digital preservation

5-vii) Access

The WPS group members were given a personal account and password, and could freely access the WPS system (Figure 1). Each participant set his/her own handle name and target body weight at the beginning.

5-viii) Mode of delivery, features/functionalities/components of the intervention and comparator, and the theoretical framework

All members were requested to fill in their present body weight and their level of lifestyle modification attained, along with their motivation level, which were expressed in a three-level scale (good [○], fair [△], and poor [×]), on the screen of the individual's system web page every week. Participants received nutritional advice and had their nutritive intakes calculated by their dietitians using a photograph of a meal. A participant and his/her dietitian could discuss their questions and comments in this personal area.

5-ix) Describe use parameters

5-x) Clarify the level of human involvement

5-xi) Report any prompts/reminders used

The WPS group members wereexpressed in a three-level scale (good [○], fair [△], and poor [×]), on the screen of the individual's system web page every week.

The EMS group members were provided with a Microsoft Excel file.They could send questions and receive nutritional advice and photo-based nutritive intakes by e-mail.

5-xii) Describe any co-interventions (incl. training/support)

The WPS group members were given a personal account and password, and could freely access the WPS system (Figure 1). Each participant set his/her own handle name and target body weight at the beginning. All members were requested to fill in their present body weight and their level of lifestyle modification attained, along with their motivation level, which were expressed in a three-level scale (good [○], fair [△], and poor [×]), on the screen of the individual's system web page every week. Participants received nutritional advice and had their nutritive intakes calculated by their dietitians using a photograph of a meal. A participant and his/her dietitian could discuss their questions and comments in this personal area.

In this system, group members could view their fellow participants' weight changes (not actual values) and their related conditions. A participant and his/her dietitian could put their queries or comments on the participant's individual screen, but the fellow participants could not write down in this column.

The EMS group members were provided with a Microsoft Excel file. They set their target body weight loss at the beginning, and subsequently filled in their present body weight, their levels of lifestyle modification attainment, and their motivation level, similar to the WPS group members. They could send questions and receive nutritional advice and photo-based nutritive intakes by e-mail. However, they could not obtain information on their fellow participants' health status.

After 12 weeks of online health support, the participants were asked to come in for remeasurement of their height, weight, waist circumference, and QOL by the same dietitian they saw at the beginning of the study.

6a) CONSORT: Completely defined pre-specified primary and secondary outcome measures, including how and when they were assessed

The primary outcome measure was change in body weight. The secondary outcome measure included changes in BMI, waist circumference, and QOL.

The comparison of changes before and after intervention between the groups was evaluated using analysis of covariance adjusted for sex, age, and the baseline value of the corresponding item at the first guidance interview. All statistical analyses were performed using JMP® 9 statistical software (SAS Institute, Inc, Cary, NC.). All tests were 2-tailed, and P values of <.05 were considered statistically significant.

6a-i) Online questionnaires: describe if they were validated for online use and apply CHERRIES items to describe how the questionnaires were designed/deployed

6a-ii) Describe whether and how "use" (including intensity of use/dosage) was defined/measured/monitored

6a-iii) Describe whether, how, and when qualitative feedback from participants was obtained

6b) CONSORT: Any changes to trial outcomes after the trial commenced, with reasons

None.

7a) CONSORT: How sample size was determined

7a-i) Describe whether and how expected attrition was taken into account when calculating the sample size

7b) CONSORT: When applicable, explanation of any interim analyses and stopping guidelines

None.

8a) CONSORT: Method used to generate the random allocation sequence

The participants were randomly assigned to either the WPS group or the EMS group using the minimization method, balancing sex (male or female), age (<40 years or =>40 years), and baseline body weight (<60 kg, 60-80 kg, or =>80 kg) by one of the authors(MA). Then they were assigned a counselor-dietitian. A total of thirteen registered dietitians under the direction of a principal dietitian provided nutritional counseling. Each dietitian was allocated to both one of the WPS group and one of the EMS group, with six to eight participants of a group being supported by one assigned dietitian during the study period. The follow-up period was 12 weeks for both groups.

8b) CONSORT: Type of randomisation; details of any restriction (such as blocking and block size)

The participants were randomly assigned to either the WPS group or the EMS group using the minimization method, balancing sex (male or female), age (<40 years or =>40 years), and baseline body weight (<60 kg, 60-80 kg, or =>80 kg) by one of the authors(MA). Then they were assigned a counselor-dietitian.

9) CONSORT: Mechanism used to implement the random allocation sequence (such as sequentially numbered containers), describing any steps taken to conceal the sequence until interventions were assigned

The participants were randomly assigned to either the WPS group or the EMS group using the minimization method, balancing sex (male or female), age (<40 years or =>40 years), and baseline body weight (<60 kg, 60-80 kg, or =>80 kg) by one of the authors(MA). Then they were assigned a counselor-dietitian.

10) CONSORT: Who generated the random allocation sequence, who enrolled participants, and who assigned participants to interventions

The participants were randomly assigned to either the WPS group or the EMS group using the minimization method, balancing sex (male or female), age (<40 years or =>40 years), and baseline body weight (<60 kg, 60-80 kg, or =>80 kg) by one of the authors(MA). Then they were assigned a counselor-dietitian.

11a) CONSORT: Blinding - If done, who was blinded after assignment to interventions (for example, participants, care providers, those assessing outcomes) and how

11a-i) Specify who was blinded, and who wasn't

None.

This study was an open prospective individual randomized controlled trial.

11a-ii) Discuss e.g., whether participants knew which intervention was the "intervention of interest" and which one was the "comparator"

11b) CONSORT: If relevant, description of the similarity of interventions

The WPS group members were given a personal account and password, and could freely access the WPS system (Figure 1). Each participant set his/her own handle name and target body weight at the beginning. All members were requested to fill in their present body weight and their level of lifestyle modification attained, along with their motivation level, which were expressed in a three-level scale (good [○], fair [△], and poor [×]), on the screen of the individual's system web page every week. Participants received nutritional advice and had their nutritive intakes calculated by their dietitians using a photograph of a meal. A participant and his/her dietitian could discuss their questions and comments in this personal area.

In this system, group members could view their fellow participants' weight changes (not actual values) and their related conditions. A participant and his/her dietitian could put their queries or comments on the participant's individual screen, but the fellow participants could not write down in this column.

The EMS group members were provided with a Microsoft Excel file. They set their target body weight loss at the beginning, and subsequently filled in their present body weight, their levels of lifestyle modification attainment, and their motivation level, similar to the WPS group members. They could send questions and receive nutritional advice and photo-based nutritive intakes by e-mail. However, they could not obtain information on their fellow participants' health status.

After 12 weeks of online health support, the participants were asked to come in for remeasurement of their height, weight, waist circumference, and QOL by the same dietitian they saw at the beginning of the study.

12a) CONSORT: Statistical methods used to compare groups for primary and secondary outcomes

We conducted intention-to-treat analyses in this study. All data are expressed as mean ± SD. BMI was defined as the weight (kg) over height (m) squared. Baseline characteristics were compared between the groups using unpaired t-test for numerical variables and chi-square test for categorical variables. The comparison of changes before and after intervention between the groups was evaluated using analysis of covariance adjusted for sex, age, and the baseline value of the corresponding item at the first guidance interview. All statistical analyses were performed using JMP® 9 statistical software (SAS Institute, Inc, Cary, NC.). All tests were 2-tailed, and P values of <.05 were considered statistically significant.

12a-i) Imputation techniques to deal with attrition / missing values

We conducted intention-to-treat analyses in this study. All data are expressed as mean ± SD. BMI was defined as the weight (kg) over height (m) squared. Baseline characteristics were compared between the groups using unpaired t-test for numerical variables and chi-square test for categorical variables. The comparison of changes before and after intervention between the groups was evaluated using analysis of covariance adjusted for sex, age, and the baseline value of the corresponding item at the first guidance interview. All statistical analyses were performed using JMP® 9 statistical software (SAS Institute, Inc, Cary, NC.). All tests were 2-tailed, and P values of <.05 were considered statistically significant.

12b) CONSORT: Methods for additional analyses, such as subgroup analyses and adjusted analyses

We conducted intention-to-treat analyses in this study. All data are expressed as mean ± SD. BMI was defined as the weight (kg) over height (m) squared. Baseline characteristics were compared between the groups using unpaired t-test for numerical variables and chi-square test for categorical variables. The comparison of changes before and after intervention between the groups was evaluated using analysis of covariance adjusted for sex, age, and the baseline value of the corresponding item at the first guidance interview. All statistical analyses were performed using JMP® 9 statistical software (SAS Institute, Inc, Cary, NC.). All tests were 2-tailed, and P values of <.05 were considered statistically significant.

RESULTS

13a) CONSORT: For each group, the numbers of participants who were randomly assigned, received intended treatment, and were analysed for the primary outcome

A total of 196 participants were recruited for this trial from July 2008 through February 2009. Among them, three participants with a BMI<24.5 at the first guidance interview were excluded, and the remaining 193 were randomly assigned to either the WPS group (97) or the EMS group (96). Ten persons from the WPS group and eight persons from the EMS group dropped out during the study period, leaving 87 in the WPS group and 88 in the EMS group to complete the study (Figure 2).

13b) CONSORT: For each group, losses and exclusions after randomisation, together with reasons

Ten persons from the WPS group and eight persons from the EMS group dropped out during the study period, leaving 87 in the WPS group and 88 in the EMS group to complete the study (Figure 2).

13b-i) Attrition diagram

14a) CONSORT: Dates defining the periods of recruitment and follow-up

Carried out from July 2008 through February 2009.

14a-i) Indicate if critical "secular events" fell into the study period

14b) CONSORT: Why the trial ended or was stopped (early)
None.
15) CONSORT: A table showing baseline demographic and clinical characteristics for each group
Baseline characteristics of the participants are shown in Table 1. Mean age was 50 years, and mean body weight was around 78 kg in both groups. There were no significant differences in sex ratio, BMI, waist circumference, target body weight loss, or QOL levels between the groups.
15-i) Report demographics associated with digital divide issues
Baseline characteristics of the participants are shown in Table 1. Mean age was 50 years, and mean body weight was around 78 kg in both groups. There were no significant differences in sex ratio, BMI, waist circumference, target body weight loss, or QOL levels between the groups.
16a) CONSORT: For each group, number of participants (denominator) included in each analysis and whether the analysis was by original assigned groups
16-i) Report multiple “denominators” and provide definitions
A total of 196 participants were recruited
16-ii) Primary analysis should be intent-to-treat

17a) CONSORT: For each primary and secondary outcome, results for each group, and the estimated effect size and its precision (such as 95% confidence interval)
Differences in changes of outcomes between the WPS group and EMS group are shown in Table 2. The loss in body weight was significantly greater in the WPS group than in the EMS group (-1.6 kg versus -0.7 kg, adjusted P=.038). The decrease in BMI was also greater in the WPS group than in the EMS group (-0.6 versus -0.3, adjusted P=.051). However, there were no significantly different changes in waist circumference. Changes in QOL scores, general health perception, vitality, role functioning related to physical and emotional problems, and mental health, were not significantly different between the groups, either.
17a-i) Presentation of process outcomes such as metrics of use and intensity of use

17b) CONSORT: For binary outcomes, presentation of both absolute and relative effect sizes is recommended
None.
18) CONSORT: Results of any other analyses performed, including subgroup analyses and adjusted analyses, distinguishing pre-specified from exploratory
None.
18-i) Subgroup analysis of comparing only users

19) CONSORT: All important harms or unintended effects in each group
None.(Health intervention study)
19-i) Include privacy breaches, technical problems

19-ii) Include qualitative feedback from participants or observations from staff/researchers

DISCUSSION
20) CONSORT: Trial limitations, addressing sources of potential bias, imprecision, multiplicity of analyses
20-i) Typical limitations in ehealth trials
This study has some inherent limitations. First, the WPS system is limited to those who can use a personal computer. Smart phones and tablet devices are currently available, and we will develop a modified WPS system that works on these devices. Second, we only observed the effect on weight loss. The true end point of nutritional counseling should be the reduction in mortality and morbidity from cardiovascular diseases. Weight loss could prevent cardiovascular events [36]. Therefore, we consider that weight change is reasonable as a short-term index.
21) CONSORT: Generalisability (external validity, applicability) of the trial findings
21-i) Generalizability to other populations

21-ii) Discuss if there were elements in the RCT that would be different in a routine application setting

22) CONSORT: Interpretation consistent with results, balancing benefits and harms, and considering other relevant evidence
22-i) Restate study questions and summarize the answers suggested by the data, starting with primary outcomes and process outcomes (use)
We developed the WPS system, which is capable of activating group dynamics, and evaluated its effectiveness in controlling body weight in a randomized controlled trial. In this trial, the WPS yielded participants with a significantly greater weight loss than did the conventional EMS.
22-ii) Highlight unanswered new questions, suggest future research

Other information
23) CONSORT: Registration number and name of trial registry
Clinical Trial Registration Information: http://www.umin.ac.jp/ctr/index-j.htm
UMIN000009147
24) CONSORT: Where the full trial protocol can be accessed, if available
This study was approved by the Ethics Committee of Kyoto University Graduate School of Medicine.
25) CONSORT: Sources of funding and other support (such as supply of drugs), role of funders
None.
X26-i) Comment on ethics committee approval
All procedures were conducted according to the Declaration of Helsinki. Participants submitted their written informed consent prior to participation. This study was approved by the Ethics Committee of Kyoto University Graduate School of Medicine.
x26-ii) Outline informed consent procedures

X26-iii) Safety and security procedures

X27-i) State the relation of the study team towards the system being evaluated
